# Building Trust and Connection: A Family Systems Perspective on Black Veteran Reintegration

**DOI:** 10.1111/jmft.70144

**Published:** 2026-05-12

**Authors:** Lastenia Francis

**Affiliations:** ^1^ Department of Human Development and Family Science, Marriage and Family Therapy Program Syracuse University Syracuse New York USA

**Keywords:** Black veterans, family systems, military reintegration, racialized stress, relational trust

## Abstract

Reintegration after military deployment is often viewed as an individual process, though it occurs in family systems. Black veterans remain underrepresented in reintegration research, particularly in studies focusing on relational trust and racialized stress. Semi‐structured interviews were conducted with 23 veterans of Operation Enduring Freedom (OEF) and Operation Iraqi Freedom (OIF) revealed four interrelated themes: presence, communication, racial identity, and family role negotiation. Findings indicate that trust was built through everyday relational processes rather than immediate emotional disclosure, and racial identity served as both a resource and a strain. These findings highlight trust as a relational process shaped by family dynamics and sociocultural contexts, extending reintegration literature.

## 1 Reintegration as a Family Systems Process

Reintegration begins when a service member returns home from deployment, but it is far more than a single event. According to Elnitsky et al. (2017), reintegration is an ongoing process that requires families to reorganize roles, renegotiate relationships, and adjust emotionally to the returning service member, affecting not only the family but also the community and work settings. Paley et al. (2013) further emphasize that these adjustments are systemic, influencing all levels of the family system. Drew et al. (2022) describe reintegration as occurring in phases—preparation, homecoming, and longer‐term stabilization—each bringing its own shifts in expectations and roles. During deployment, families adjust by shifting caregiving responsibilities, decision‐making, and daily routines. Upon return, these patterns require careful renegotiation to fit the family's new reality, rather than a simple reversal (Pincus et al. 2001). Thus, reintegration disrupts family equilibrium and calls for the entire system to recalibrate (Elnitsky et al. 2017; Paley et al. 2013).

A family systems perspective frames reintegration as a relational restructuring that affects the entire family system (Brennan et al. 2021; Paley et al. 2013). Couples must navigate changes in intimacy and relational roles; parents adapt their approaches to hierarchy and co‐parenting; and children adjust to evolving family structures and expectations (Riggs and Riggs 2011). Because these subsystems are interconnected, stress or disruption in one relationship can ripple throughout the family. O'Neal and Mancini (2020) found that reintegration challenges, especially conflict between parents, can impact adolescent adjustment, highlighting the mutual influence among family processes during this period. Family systems theory thus conceptualizes reintegration as a set of ongoing, interactive processes where each member's experiences shape the responses of others (Paley and Hajal 2022).

Role renegotiation and emotional regulation are central to family reintegration. Service members often return to families that have developed new routines during their absence, which can create uncertainty and feelings of exclusion (Knobloch and Theiss 2012). Flexible roles and open communication are crucial for adapting to these changes, as families must maintain and adjust new family patterns over time (Glorieux et al. 2023). Communication shifts from the initial emotional intensity of reunion to the practical work of daily coordination and support (Knobloch‐Fedders et al. 2020). When families experience stress during reintegration, parents' post‐traumatic stress disorder (PTSD) symptoms, not the number or length of deployments, are directly linked to poorer child adjustment, with maternal PTSD symptoms also undermining effective parenting (Gewirtz et al. 2018). Sandoz et al. (2015) found that psychological flexibility, or the ability of family members to adapt their behavior and communication to changing demands, is critical for buffering negative outcomes and supporting healthier family adjustment after deployment. Ultimately, the outcome of reintegration depends on the family system's ability to reorganize roles, regulate emotions, and build a new sense of balance together.

### 1.1 Trust as a Relational Process During Reintegration

Within the context of reintegration, trust is a dynamic process that develops through ongoing interactions and mutual reliance, rather than existing as a fixed trait (Melvin et al. 2015). As families navigate postdeployment uncertainty about the partnership, commitment, or emotions, can create space for doubt and foster negative interpretations if not managed (Knobloch et al. 2021). Open communication and honest disclosure reduce ambiguity and build shared understanding, thereby strengthening relational bonds (Drew et al. 2022; Knobloch et al. 2023).

Building and sustaining trust during reintegration depends on communication that promotes both vulnerability and emotional safety. Couples strengthen trust by sharing emotions, acknowledging mistakes, and repairing relational ruptures by apologizing and reconnecting (Knobloch et al. 2023; Melvin et al. 2015). Gradual adjustment, moderated expectations, and patience help reduce defensiveness and foster trust (Adler et al. 2011). Blow et al. (2015) argue that emotionally focused therapy is an effective model for addressing relationship difficulties in military couples dealing with PTSD, as it emphasizes restructuring emotional responses and fostering secure attachment bonds. Over time, consistent reliability and responsiveness build a sense of stability and make trust a dynamic, ongoing process within the family system (Kopacz et al. 2018).

Trust within the family system can erode when interactional patterns create ambiguity or emotional risk. For example, suspicion, particularly if one partner believes information is being withheld, heightens uncertainty and undermines trust even in the absence of clear betrayal (Knobloch et al. 2021). Emotional withdrawal, limited disclosure, or stonewalling signals unavailability, which partners may interpret as a lack of care or commitment. Knobloch‐Fedders et al. (2020) found that military couples experience significant and fluctuating shifts in closeness, communication, and relational uncertainty throughout reintegration as they adjust to postdeployment life. Perkins (2025) highlights that racism can lead to protective withdrawal and guardedness in Black couples, further discouraging emotional openness. Trust may also be threatened when the at‐home partner's competence is challenged, or when authority is quickly reclaimed after deployment, prompting resentment and distrust (Adler et al. 2011).

### 1.2 Racialized Stress, Intersectionality, and Black Veteran Family Reintegration

Racial hierarchies embedded in military organizations profoundly shape the experiences of Black service members, creating daily challenges (e.g., harassment, limited mentorship, and barriers to advancement) with consequences that extend beyond military careers (Cancelmo et al. 2025). Persistent racial prejudice within the armed forces has lasting impacts, including increased vulnerability to trauma (Burk and Espinoza 2012). Dohrenwend et al. (2008) found that Black Vietnam veterans experienced PTSD at higher rates than White peers, with racism inside and outside the military directly contributing to this disparity. Sohn and Harada (2008) demonstrated that racial discrimination is linked to poorer health outcomes for minority veterans. Reintegration for Black veterans requires healing from both military and racial trauma, as these wounds intersect and compound (Winters et al. 2023). This dual burden strains family relationships, as protective hypervigilance can hinder emotional openness.

Intersectionality, as defined by Crenshaw (1989) and expanded by Collins (2019), is more than a theoretical concept; it is a lived reality that shapes Black veterans' reintegration, exposing the limitations of race‐neutral frameworks. Burk and Espinoza (2012) document the history of Black military service is marked by exclusion, segregation, and inequitable benefits, while Carlson et al. (2018) argue that this legacy fuels mistrust and makes homecoming a negotiation with systemic inequity and collective memory. These complexities distinguish Black veterans from broader analyses of “service members of color” and highlight the need for focused research. Racialized stress compounds trauma. Coleman (2016) found that Black veterans with high combat exposure experience more severe PTSD than White and Hispanic peers. McClendon et al. (2021) demonstrate that for Black women veterans, discriminatory stress at the intersection of race and gender strongly predicts increases in PTSD severity. Winters et al. (2023) found that Black male veterans' racial and military identities interact to shape their postdeployment reintegration experiences and mental health.

Black veterans' reintegration is shaped not just by transition logistics but by the lived realities of systemic racism, institutional bias, and relentless racialized stress. Perkins (2025) describes how discrimination is absorbed and embodied, affecting both stress responses and intimate relationships. Burk and Espinoza (2012) document the ongoing exclusion and barriers Black veterans encounter when returning to civilian life, while Cancelmo et al. (2025) highlight the impact of institutional bias, especially in administrative separations. Winters et al. (2023) show that accumulated racism fosters hypervigilance and undermines trust, both of which are vital for successful reintegration. Whealin et al. (2024) find that systemic racism's psychological toll may intensify after military service, compounding deployment‐related trauma. Black veterans face amplified service burdens due to ongoing racialized harm. Cancelmo et al. (2025) further identify structural disparities, such as disproportionate Other Than Honorable (OTH) discharges, limited benefits, and strained family life. Financial precarity, healthcare barriers, and institutional mistrust disrupt family communication, trust, and emotional climate.

Reintegration for Black veterans and their families is complicated by structural inequities and exclusionary narratives. Nillni et al. (2023) found that discrimination in work, housing, health care, and public life operates as a daily psychosocial stressor, fueling mental health disparities among veterans of color. Burk and Espinoza (2012) document how the legacy of segregation, lack of recognition, and limited advancement in Black military service reflect broader patterns of racial stratification. Carlson et al. (2018) and Williams‐Washington and Mills (2018) further highlight how these inequities are embedded in American institutions and shape lived experiences. Perkins (2025) shows that microaggressions and racialized encounters at work disrupt family dynamics by influencing emotional expression, escalating conflict, and undermining feelings of safety at home. These ongoing challenges are compounded by historical and intergenerational trauma (e.g., slavery, Jim Crow segregation Laws, mass incarceration), as racial trauma endures across generations due to chronic exposure to systemic harm (Cénat 2023; Coleman 2016).

Coleman (2016) and Lee et al. (2023) demonstrate that intergenerational trauma transmits racialized stress across generations, shaping care, trust, and emotional boundaries with Black families. Carlson et al. (2018) emphasize that these adaptations, rooted in survival and love, transform how family support is given and received. However, Knobloch et al. (2023) note that most reintegration research focuses on White families, leaving Black experiences less explored. Too often, race is treated as a checkbox rather than as a structural force in reintegration. Dorsey Holliman et al. (2018) highlight that much scholarship centers on pathology and neglects the resilience found in spirituality, kin networks, and racial identity, resources vital to Black veterans' well‐being. As a result, conventional frameworks overlook how structural racism and intersectional stress shape Black veteran families. Centering these realities moves us beyond race‐neutral paradigms to reflect the complex stories of Black veterans and their families.

In summary, the literature highlights that reintegration is a complex process that affects the whole family (Adler et al. 2011; Elnitsky et al. 2017; Paley et al. 2013), requiring ongoing adjustment of roles, emotions, and relationships (Drew et al. 2022; Knobloch‐Fedders et al. 2020; Riggs and Riggs 2011). Trust is central, growing through communication, vulnerability, and responsiveness, but can be easily harmed by uncertainty, withdrawal, or changes in power or safety (Knobloch et al. 2021; Knobloch et al. 2023; Melvin et al. 2015). For Black veterans and their families, these challenges are made harder by racialized stress, systemic inequities, and intersecting identities (Burk and Espinoza 2012; Carlson et al. 2018; McClendon et al. 2021; Perkins 2025; Winters et al. 2023). While past research has looked at reintegration, trust, and racialized experiences as separate topics, we know little about how they come together in the lives of Black veteran families. It is unclear how racialized stress and military experiences together shape trust and connection during reintegration. This study aims to address this gap by exploring how Black veterans and their families experience reintegration and how it affects trust and connection in their family life.

## 2 Method

### 2.1 Research Design

This study used a constructivist grounded theory approach to explore the reintegration experiences of Black veterans after deployment, emphasizing relational trust, communication, role transitions, and meaning‐making in family and social contexts. Constructivist grounded theory emphasizes the co‐construction of meaning between the researcher and participants' lived experiences and considers the sociocultural and institutional factors shaping those realities (Charmaz 2025). While the original aim was to involve both veterans and their family members to examine reintegration as a relational process, most veterans were reluctant to include relatives, limiting family participation. Thus, the analysis focused primarily on veterans' accounts, with themes of relational distance, emotional withdrawal, and barriers to trust and engagement emerging.

### 2.2 Participants and Recruitment

The sample comprised 23 Black military veterans who served during Operation Enduring Freedom (OEF) or Operation Iraqi Freedom (OIF), prior to 2020. Participants were aged 25 to 59 years, with the majority between 25 and 39 years. Fourteen participants served in the Army, five in the Marine Corps, and four in the Navy. Self‐identified backgrounds included African (*n* = 3), African American (*n* = 8), and African Caribbean/Afro‐Caribbean (*n* = 12). Family structures among participants included partnered (married/domestic partner; *n* = 10, but only nine cohabitated with their partner), parenting (children in household; *n* = 8), extended family (multiple adults, nonpartner; *n* = 7), and independent living (*n* = 4) arrangements. These configurations were considered contextual influences on reintegration, not variables for direct comparison. Three family members participated in supplementary interviews; however, these data were excluded from thematic analysis to maintain analytic focus on veterans' lived experiences. All participants had a prior diagnosis of PTSD from the Department of Veterans Affairs, although symptom severity was not an inclusion criterion. Participants were recruited through Veterans Affairs programs in New York City, social media outreach, and snowball sampling. All identifying information was anonymized to ensure participant confidentiality. A summary of veteran participant demographics, including age, service branch, racial/ethnic identity, and education, is presented in Table 1.

**Table 1:** Participant characteristics (*N* = 23 veterans).

Characteristic	*n*	%
Age (years)		
25–39	15	65.2
40–59	8	34.8
Branch of service		
Army	14	60.9
Marine corps	5	21.7
Navy	4	17.4
Race/ethnicity		
African	3	13.0
African American	8	34.8
African Caribbean/Afro‐Caribbean	12	52.2
Education		
High school diploma/GED	2	8.7
Some college	2	8.7
Associate's degree	3	13.0
Bachelor's degree	9	39.1
Master's degree	7	30.4

*Note:* Demographic data are based on 23 veteran participants; family members (*n* = 3) were excluded from the demographic surveys and are therefore not included in this table. Participant 12 (a veteran's wife) was removed entirely, but the pseudonyms remained unchanged.

### 2.3 Data Collection

In 2020, semi‐structured interviews were conducted for 60 to 90 min, either in person or virtually, at the participant's preference. The interview protocol explored reintegration, racial and cultural identity, and family dynamics, with a focus on relational trust and connection (see Appendix SA for the full interview guide). Interviews began with, “Tell me about your reintegration experiences after deployment,” allowing participants to define reintegration in their own terms. Follow‐up questions explored changes in relationships, support, and strain within the family. Probing questions included: “What has felt different in your relationship since reintegration?” “What does feeling connected mean to you?” “What helps you feel supported?” and “What makes it easier or harder to lean on someone?” As participants highlighted themes of relational distance, dependability, and reconnection, additional probes about trust and connection were added iteratively. Thus, trust became a focus through participants' narratives rather than a predetermined analytic category.

To address the focus on Black veterans, the interview guide included questions on racial identity and military experiences, such as, “When did you first become aware of your race, and how does it shape your roles in your family (e.g., partner, parent)?” and “How do you see the influence of your race on your military experiences?” Additional prompts covered emotional availability, communication, and role transitions postdeployment. Although race was not initially an analytic category, participants often described discrimination, underscoring its relevance to trust and connection. All interviews were audio‐recorded, transcribed verbatim, de‐identified, and supplemented with field notes. Institutional Review Board approval and informed consent were obtained.

### 2.4 Researcher Positionality

The author is a Black woman and a licensed marriage and family therapist with clinical experience serving veteran populations. Her background shaped the recruitment, data collection, and analysis. Some veterans were initially hesitant to participate, reflecting broader mistrust of research within Black communities (George et al. 2014; Henderson et al. 2024; Scharff et al. 2010; Tamlyn et al. 2023). Participation increased due to shared racial identity and peer referrals, illustrating how common backgrounds foster trust (Moore et al. 2023). Reflexive practices were used to examine the influence of the author's identity and training on coding and theory development, supporting qualitative standards and enhancing analytic credibility (Berger 2015).

### 2.5 Data Analysis

Data analysis followed Charmaz (2025) constructivist grounded theory procedures, beginning with line‐by‐line coding to capture participants' language, then focused coding to group significant codes into higher‐order categories. Theoretical coding defined relationships among trust, relational withdrawal, and reintegration. Memo writing and constant comparison documented insights and refined categories over time (Birks et al. 2008). During the analytic process, data from family members were initially incorporated. However, because only three family members participated and to preserve the study's focus on veteran experiences, these data were excluded from the primary thematic structure. The data set was then re‐examined to ensure the themes remained grounded in veteran narratives, resulting in a refinement of the thematic emphasis. Analysis continued until theoretical sufficiency was achieved, indicated by the absence of new conceptual insights from additional data (Charmaz 2025).

### 2.6 Trustworthiness and Rigor

Several strategies enhanced trustworthiness. Member checking with a subset of participants, who reviewed and commented on preliminary interpretations, helped support the credibility of the findings (Birt et al. 2016). Additionally, peer debriefing with colleagues experienced in qualitative and family systems research strengthened analytic rigor. An audit trail documented coding decisions and analytic shifts, ensuring dependability and confirmability (Birks et al. 2008). Reflexive journaling was also maintained to examine how the researcher's positionality influenced interpretation (Berger 2015).

## 3 Findings

The findings indicate that Black veterans and their families established trust during the reintegration process through continuous relational interactions rather than through isolated events. Four interconnected themes emerged: presence, communication, racial identity, and family role negotiation. These themes illustrate how trust can be shaped, strained, and rebuilt over time. Trust was influenced by emotional availability, relational attunement, shared meaning‐making, and adaptation of roles within family systems.

### 3.1 Theme 1: Presence—“Being There”

Participants described trust as something rebuilt gradually through consistent relational patterns rather than restored through a single moment. Trust was shaped by recurring experiences of emotional availability and reliability, whether family members were consistently “there” in ways that felt steady and dependable. When presence was experienced as predictable and engaged, relational safety strengthened. Conversely, emotional inconsistency or perceived absence intensified isolation and reinforced withdrawal. Veterans explained that patterns of presence and absence directly influenced their willingness to re‐engage, with sustained availability fostering connection and inconsistency prompting self‐protective distance.

#### 3.1.1 Consistency in Presence

Veterans identified consistency as a stabilizing force during reintegration, emphasizing that regular communication and predictable outreach reduced isolation and fostered trust. Ongoing contact, described as “always communicated” and “talked almost every day,” helped sustain relational safety even amid emotional numbness. For some, this consistency began during deployment and continued after return. Participant 23 reflected on his mother's steady contact throughout deployment and homecoming, explaining, “She wanted to ensure I did not feel unloved.” Similarly, Participant 3 described his mother's persistent engagement despite her disapproval of his service and her own health struggles: “She consistently asks, ‘What are you doing today? Why not come over?’”

For others, the value of consistency became clearer over time, particularly following periods of withdrawal. Participant 5 initially experienced his cousin's repeated outreach as intrusive but later recognized it as support: “At first, I found it annoying… I didn't appreciate it until recently. I didn't realize they were actually supporting me.” Several veterans described distancing themselves as a response to trauma while simultaneously benefiting from others' reliable presence. Participant 10 acknowledged, “My wife says I don't open up to her enough… So I distance myself.” Yet, he emphasized, “I struggled and succeeded after the military largely because my family was there for me… They were just there.” Participant 13 similarly noted that trust was strengthened through proactive check‐ins: “I like the fact that my girlfriend will reach out to me during the week to check in.”

#### 3.1.2 Inconsistency in Presence

Trust declined when relationships were inconsistent, emotionally misaligned, or seen as unreliable. Veterans reported “no support,” feeling “ignored,” “rejected,” or “socially isolated,” especially when initial engagement was not sustained. Participant 9 noted that brief outreach undermined trust: “Even if they initially came around, they didn't stay long enough for me to trust them.” Participant 11 described support that was present but distant, complicating relational safety: “They were there, but they expected me to handle it myself.” These experiences encouraged self‐reliance and withdrawal instead of vulnerability.

Trust eroded further when support felt conditional or decreased during distress. Participant 17 reflected, “It just seemed like everyone was giving up on me, pushing me to see problems before I was ready.” Participant 18 also described unmet expectations in her marriage: “I want more support from my husband on this issue, but I am not getting it.” For Participant 20, ongoing absence led to feeling “ignored and rejected” and “socially isolated,” which resulted in protective withdrawal over time. Inconsistency did not just weaken connection; it changed expectations of availability, reinforced guardedness, and limited trust restoration during reintegration.

### 3.2 Theme 2: Communication—“Being Heard”

Participants identified communication as essential to relational safety during reintegration. Veterans noted that tone, responsiveness, and emotional attunement were more important than frequency. Respectful and emotionally safe exchanges encouraged reconnection, while dismissive or misaligned responses led to withdrawal and increased emotional distance. Communication was a key factor influencing veterans' willingness to stay engaged in their relationships.

#### 3.2.1 Supportive Communication

Veterans characterized supportive communication as demonstrating respect, understanding, and emotional awareness, while avoiding any sense of pressure to disclose personal experiences. They expressed a preference for conversations that aligned with their individual readiness to engage and proceeded at a comfortable pace. Participant 1 emphasized that developing effective communication required time:

I believe we've been able to cope by learning from each other… Even though it took three months for me to communicate my feelings, she was glad I verbalized them. A key part of our coping mechanism is open communication and understanding.

Participant 23 prioritized expressions of care over being subjected to excessive questioning: “We would communicate and pray together… They checked in and made sure I was comfortable.” For Participant 4, supportive communication entailed being treated as capable and independent, rather than as fragile or impaired. He expressed frustration when others acted with excessive caution or interpreted directness as evidence of psychological harm, stating, “People often assume that being abrasive means war has damaged you… Just let me do my job without being treated like I should take it easy. I'm not soft. I've realized that's unnecessary.” Participant 8 reported feeling encouraged by family support: “My family has been concerned about my readjustment challenges and has been very supportive, cheering me on as I make progress in therapy.” For Participant 15, support was defined by the absence of pressure: “The people who were closest to me didn't push me to discuss everything. Just being there and talking about normal things helped more than people realize.” Collectively, veterans described supportive communication as “open,” “understanding,” “comfortable,” and “grounded.” They reported feeling heard when others responded with respect and emotional awareness, without expecting them to share more than they were prepared to disclose.

#### 3.2.2 Unsupportive Communication

Veterans described unsupportive communication happening when others were emotionally absent, made assumptions, or showed little interest in their inner experiences. Instead of encouraging them to share, these interactions made them feel dismissed or ignored, which increased their sense of isolation during reintegration. Participant 6 described unsupportive communication as emotional indifference in his marriage, especially when it came to his military experiences:

I haven't received support from anyone. My wife doesn't care about my military experiences. I get no support from her… I've felt alone, especially since the person who would care the most has died.

For Participant 2, unsupportive communication meant others made assumptions without trying to understand him. He felt his family members projected their own views instead of listening to his:

I didn't receive support from my girlfriend; she was just focused on getting pregnant. She wasn't supportive. My mother also wasn't a support system. My brother and I never got along. He assumes he knows what someone is going through, but you can't truly understand another's experience unless you walk in their shoes. I mostly kept to myself. I didn't trust anyone, and I didn't care about others' opinions.

Participant 7 associated unsupportive communication with feeling invisible and emotionally neglected in his family: “I get angry easily. I don't enjoy spending time with my family. My marriage is strained. At home, no one truly sees me. My wife and I hardly communicate; she's consumed with issues related to the coronavirus.”

Even when others showed concern, a lack of real understanding could still hurt trust. Participant 8 described communication that focused more on reassurance or wanting things to go back to normal, rather than understanding how he had changed inside:

They don't think they truly understand what I've been through. They can't accept that I'm different. They want me to improve and return to who I was…I keep them at a distance because, despite their support, they couldn't handle everything I need to express.

Overall, unsupportive communication was not just about what was said or left unsaid, but about a lack of real understanding. When veterans felt misunderstood, dismissed, or pushed to act like their old selves, they often became emotionally distant to protect themselves.

### 3.3 Theme 3: Racial Identity—“Being Black”

During the reintegration process, racial identity influenced how veterans navigated trust within their families, especially regarding decisions about disclosing experiences of racism encountered during military service. Participants identified racial identity as both a source of resilience and meaning, as well as a context that increased vigilance and emotional guardedness. A central tension emerged concerning whether acknowledging racial harm fostered connection or introduced risk within family relationships. Overall, racial identity operated as a dynamic factor shaping trust, communication, and belonging throughout reintegration.

#### 3.3.1 Racial Identity As a Resource

For many veterans, racial identity served as a resource that provided meaning, orientation, and relational grounding during reintegration. Rather than being peripheral to family life, it influenced how veterans interpreted stress, defined responsibility, and extended care within their households. Participant 24 reflected, “My dad used to tell me that because we were Black, we had to work twice as hard. I didn't understand it until I was in the military, and even more so now as I'm raising my son. I worry every day—if he can't behave in school, will he be treated like the other Black boys who ended up dead? That fear shapes how I parent and how I reintegrate.” Although rooted in concern, this racial consciousness provided a framework for intentional parenting and vigilance, reinforcing a commitment to presence and protection within the family. In this way, racial identity offered structure and purpose amid the instability of postdeployment adjustment.

Other participants described racial identity as a source of continuity and shared understanding that strengthened relational bonds. Participant 2 emphasized that “knowing my history” helped him contextualize both his military experiences and his daughter's future, while Participant 18 connected her service to her father's experiences of discrimination as a World War II veteran, noting, “It feels like it hasn't changed.” These narratives positioned racial identity as an intergenerational anchor, linking past and present in ways that informed family meaning‐making. Shared racial identity also enhanced emotional safety in intimate relationships. Veterans reported feeling more “understood” and “less burdened” to explain racial experiences when partners shared their racial background. Participant 1 explained that his wife's identity as a Black woman “increased her sensitivity to discrimination” he faced, and Participant 10 felt “safe” discussing workplace racism with his wife without needing to contextualize it. Collectively, these accounts indicate that racial identity functioned as a relational bridge, supporting trust, emotional accessibility, and cohesion during reintegration.

#### 3.3.2 Racial Identity as a Strain

For some veterans, racial identity heightened vigilance and complicated the development of trust during reintegration. Rather than providing a sense of stability, racial identity prompted increased scrutiny of others' intentions and restricted emotional openness within families. Participant 21 explained, “I didn't really think of my race as bad until I came to the United States, where I experienced harassment because of my Haitian accent and the color of my skin. It became harder to trust people, even family, when I returned because I was always second‐guessing their motives.” This account illustrates how racialized experiences outside the home can influence intimate relationships, rendering reintegration a continuous evaluation of safety rather than a straightforward return to familial closeness.

Other participants described racial identity as a source of heightened fear and diminished emotional security. Participant 16 shared,

Being Black… it feels like it has never been good enough, even for my family when I was growing up. And now, coming back, it's still not good enough. I see it in the news every day—Black people dying for no reason. Some days I don't want to be Black. I'm more afraid of dying here than I was while in Afghanistan.

For this participant, racial identity was associated with a persistent sense of threat, which complicated the perception of home as a safe environment. This strain was intensified for veterans with multiple marginalized identities. Participant 20 described being “harassed because I was Black, because I was a woman, because I was educated” and carried these intersecting experiences of invalidation into relationships where emotional support was inconsistent.

Racial differences within intimate partnerships further constrained trust. Participant 18 stated, “It's been hard, especially when I have someone who doesn't know what it means to be Black. He doesn't understand what I go through in different settings. He doesn't see racism because he's White.” Similarly, Participant 3 reflected, “She didn't really understand what it was like to be Black because she looked more White. It was hard to connect.” In these instances, anticipated misunderstanding diminished openness and reinforced emotional restraint. Even when veterans attempted to minimize the significance of race as a coping strategy, the underlying strain persisted. Participant 13 noted, “I don't really see race; they do… I didn't want to be defined by my race. I wanted to be honorable,” while Participant 14 acknowledged, “I don't think I can escape any of those identities… The news reminds me of that every day.” Collectively, these accounts indicate that racial identity constrained trust not due to individual shortcomings, but because persistent racialized threats rendered vulnerability risky, even within familial relationships.

### 3.4 Theme 4: Family Role Negotiation—“Being Needed”

Reintegration was characterized as a period of renegotiating family roles, rather than a straightforward resumption of previous responsibilities. Military service influenced expectations regarding hierarchy, authority, and structure, whereas family systems frequently adapted in the absence of the service member. Upon returning, veterans often faced conflicting assumptions about leadership, caregiving, and decision‐making. The degree of flexibility or rigidity in these role negotiations played a critical role in shaping trust, emotional safety, and opportunities for relational repair during reintegration.

#### 3.4.1 Role Flexibility

Role flexibility emerged as a primary mechanism for rebuilding trust and connection during reintegration. Instead of reverting to predeployment hierarchies, families reported engaging in shared leadership, redistributing responsibilities, and adapting decision‐making processes in response to fluctuating emotional capacities. Trust was enhanced when authority shifted fluidly, without undermining individual identity or respect. For example, Participant 19 demonstrated shared responsibility by stating, “When I'm angry, I overreact, which is why I allow my wife to discipline our children.” This willingness to relinquish disciplinary control indicated mutual respect for evolving capacities and a prioritization of family stability over rigid role expectations. Similarly, Participant 14, who was married to another veteran, described alternating leadership roles depending on which partner was experiencing mental health challenges, explaining that when her wife was having an episode, she would “take the lead in decision‐making,” and vice versa. This reciprocal arrangement exemplified collaborative adaptation rather than adherence to a fixed hierarchy.

For some veterans, role flexibility entailed redefining caregiving identities and accepting influence from family members. Participant 2 described how fatherhood disrupted patterns of anger that followed military discrimination, stating, “My daughter's arrival forced me to change… If she brought home a man like me… I'd be ashamed.” In this case, trust was re‐established through accountability and behavioral change in response to familial needs. Similarly, Participant 7 identified fatherhood as a stabilizing force during periods of PTSD‐related distress, noting, “Being a father is probably the most important thing to me… Having two boys keeps me grounded,” despite ongoing challenges in his marital relationship. Emotional role expansion further indicated flexibility; for instance, Participant 3 intentionally learned to express affirmation, stating, “I had to learn to call my daughter pretty… so she wouldn't look for it elsewhere.” Participant 16 acknowledged the importance of his partner's concern in motivating him to seek care: “If she weren't worried, I'm not sure I would have taken my PTSD seriously.” Collectively, these accounts demonstrate that trust was reinforced when families embraced vulnerability, encouraged feedback, and adapted roles collaboratively following absence or injury. Flexibility, rather than rigidity, facilitated relational repair.

#### 3.4.2 Role Strain

Role strain was evident when veterans were required to adopt stabilizing or authority‐based roles without receiving reciprocal support, which restricted opportunities for vulnerability and diminished trust. In contrast to flexible role redistribution, these patterns involved rigid or involuntary role assignments that exacerbated emotional isolation. Participant 1 illustrated this dynamic by describing overcompensation for the loss of military authority following injury:

Coming back injured, they no longer saw me as a leader, just a broken Black guy. At home, this translated into me overcompensating, being stricter and more aggressive with my wife and son, because I felt I had to hold onto some authority somewhere.

In this context, diminished institutional status increased the pressure to reassert authority within the family system, thereby reinforcing control at the expense of connection.

For some participants, role strain manifested as an intergenerational phenomenon. Participant 13 recounted being assigned a father‐figure role prior to reintegration and feeling confined to this position upon return: “When I came back, I felt like I was taking care of everyone else… I was forced to be the father figure for my family. I hated my dad for putting me in this role. I wasn't ready.” His resentment toward his father and sense of premature responsibility exemplified parentification rather than collaborative adaptation. The lack of emotional support persisted into his adult relationships, as he described seeking connection through an extramarital affair because he “didn't feel like I have anywhere else to turn.” The persistent expectation that he provide stability for others, without receiving support himself, limited trust and intimacy. Similarly, Participant 8 described a continued reversal of roles with his father: “Since returning home, I feel like I'm primarily supporting my parents… my dad appearing more like the child.” His confusion when his father attempted to check on him, “Why is he worried about me now? … It was confusing, insulting, and felt awkward,” illustrated how entrenched caregiving expectations undermined mutuality. Instead of fostering shared leadership, these dynamics imposed unilateral responsibility. Collectively, these accounts demonstrate that rigid or imposed roles restricted opportunities for vulnerability and reciprocal care, thereby weakening emotional safety during reintegration.

## 4 Discussion

**Figure 1:**
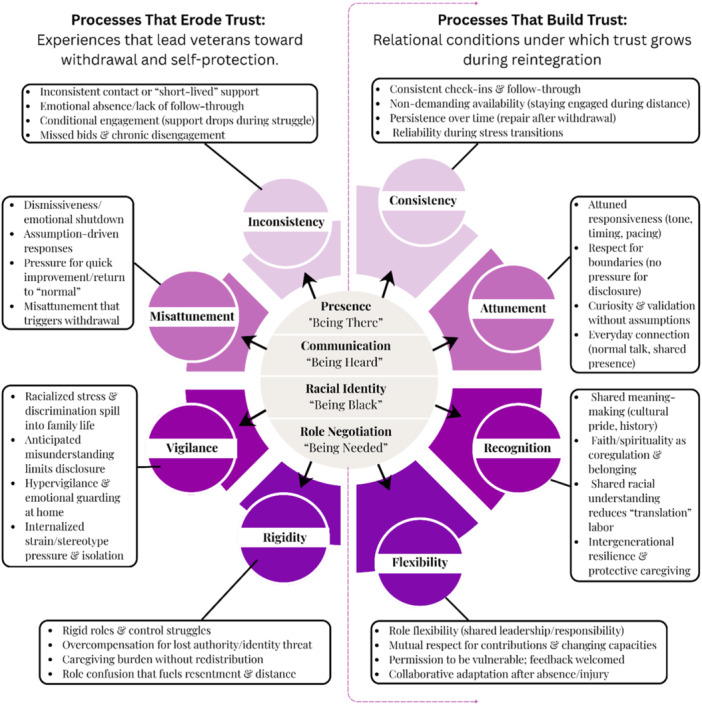
Systemic processes that build and erode trust during Black veteran reintegration. The center of the figure represents four relational domains, while outer elements depict processes that either support trust‐building (right) or contribute to trust erosion (left) during reintegration. Directional arrows indicate movement toward engagement or withdrawal. [Color figure can be viewed at wileyonlinelibrary.com]

This study examined how Black veterans and their families experience reintegration following deployment, focusing on the systemic processes that shape relational dynamics. Figure 1 illustrates how trust, communication, role flexibility, and cultural identity interact to either strengthen or strain family connection during reintegration. These interrelated processes underscore that reintegration is not an individual adjustment but a family‐level negotiation influenced by cultural, relational, and sociopolitical contexts.

### 4.1 Presence: Trust as a Mechanism for Systemic Stabilization Over Time

Presence acted as a stabilizing factor within the broader systemic changes associated with reintegration, corroborating family systems research that characterizes this period as one of blurred boundaries and role confusion (Paley et al. 2013; Pincus et al. 2001; Riggs and Riggs 2011). This study extends the findings of Knobloch‐Fedders et al. (2020), who found that emotional closeness fluctuates during postdeployment adjustment. Participants in this study described trust as developing through repeated demonstrations of availability rather than through single emotional events. This process aligns with the Military Family Stress Model (Gewirtz et al. 2018), which identifies relational reliability as essential for systemic stabilization, dyadic adjustment, and effective parenting in the context of deployment‐related stressors such as parental PTSD. Importantly, while much existing literature emphasizes the importance of emotional intensity in restoring intimacy (Sandoz et al. 2015), the current findings suggest that predictable presence, rather than immediate reciprocity, anchors trust during reintegration, echoing and extending the insights of previous longitudinal work.

These findings challenge symptom‐centered interpretations of withdrawal, which have been prominent in the literature linking PTSD symptoms to relational strain and decreased engagement (Brennan et al. 2021; Diehle et al. 2016; Renshaw et al. 2008). While prior studies often frame withdrawal as a barrier to intimacy, the present study aligns with Knobloch and Theiss (2012) and Paley and Hajal (2022) in viewing withdrawal as a regulatory strategy influenced by vigilance, grief, and emotional overload. Importantly, this study extends previous models by showing that trust is not necessarily eroded by withdrawal itself, but by inconsistencies or conditionality in presence during periods of withdrawal. Families that tolerated asymmetry in engagement were able to rebuild trust incrementally, highlighting the centrality of sustained presence over immediate emotional access in the reintegration process.

### 4.2 Communication: Trust, Attunement, and Emotional Pacing

Communication shaped trust not through frequency or skill proficiency, but through attunement and emotional pacing, a finding that builds on and extends existing research on postdeployment relational maintenance (Knobloch et al. 2023). Studies by Wen et al. (2020) and Sayers (2011) have emphasized the significance of supportive communication for military couples, noting that responsiveness and containment facilitate engagement and reduce distress. Consistent with these models, veterans in this study valued communication characterized by respect, responsiveness, and containment, which allowed engagement without pressuring vulnerability. Attachment‐oriented approaches (Riggs and Riggs 2011) and systems‐based frameworks (Paley and Hajal 2022) have long highlighted the importance of emotional responsiveness and availability during reintegration. The present findings corroborate these frameworks, showing that when communication was emotionally attuned and paced according to veterans' readiness, it fostered relational safety and sustained engagement. Conversely, emotionally absent or assumption‐driven communication, identified as a barrier to intimacy in Knobloch‐Fedders et al. (2020), was associated with withdrawal and reduced disclosure in this sample as well.

Extending beyond prior literature that frequently frames communication as a set of teachable skills (Blow et al. 2015; Sayers 2011; Wen et al. 2020), this study demonstrates that veterans experienced communication primarily as a measure of relational safety. This aligns with systems‐oriented models of emotion regulation (Paley and Hajal 2022), which emphasize co‐regulation within the family system, and with Knobloch and Theiss (2012), who found that communication effectiveness depends on mutual accessibility and the ability to re‐engage after periods of silence. In this context, silence did not always indicate disconnection; instead, it sometimes served as a regulatory pause, echoing findings from Knobloch and Theiss (2012), so long as mutual accessibility (i.e., remaining physically present, signaling intention to return to the conversation, maintaining soft tone or eye contact) and re‐engagement remained possible. However, prolonged emotional unavailability (i.e., emotional shutdown, rigidity, or prolonged disengagement that leaves the family member uncertain about availability and relational safety), consistent with evidence from Knobloch‐Fedders et al. (2020), signaled withdrawal and eroded trust. Thus, these findings both affirm and extend prior models by underscoring the central role of relational safety, emotional pacing, and co‐regulation in effective communication during reintegration for Black veteran families.

### 4.3 Racial Identity: Trust Within Ongoing Racialized Contexts

Racial identity played a dual role in influencing trust, disclosure, and connection during the reintegration process. This finding is consistent with prior research documenting the significance of racial identity as both a protective resource and a source of vulnerability for Black veterans and their families (Dorsey Holliman et al. 2018; McClendon et al. 2021). Participants indicated that their racial identity provided cultural meaning, pride, and a sense of shared understanding, echoing findings by Carlson et al. (2018) on the buffering effects of cultural resources, while also increasing vigilance and emotional guardedness, as described in research on minority stress and racial trauma (Nillni et al. 2023; Whealin et al. 2024). The tension participants described, regarding whether discussing experiences of racialized harm within family relationships felt safe or burdensome, aligns with work framing racialized harm as cumulative, intergenerational, and ongoing (Cénat 2023; Sharifian et al. 2024).

Consistent with studies demonstrating that discrimination and sociocultural inequities shape trauma exposure and mental health consequences beyond combat experiences (McClendon et al. 2021; Nillni et al. 2023; Whealin et al. 2024), this study emphasizes that reintegration is embedded within persistent racialized contexts. While much existing literature focuses on individual symptom trajectories and disparities (Carlson et al. 2018; Sharifian et al. 2024; Winters et al. 2023), our findings extend this work by broadening the lens to examine how racial identity and racialized stress are navigated within family systems, affecting relational safety and trust (Dorsey Holliman et al. 2018). Specifically, shared racial identity within families often diminished the need for explanation and facilitated emotional connection, consistent with research on cultural buffers (Carlson et al. 2018). In contrast, racial differences within partnerships could limit disclosure due to concerns about misunderstanding or invalidation, prompting self‐protective strategies, extending previous findings on minority stress and relational dynamics (Cénat 2023; Sharifian et al. 2024).

### 4.4 Family Role Negotiation: Trust, Authority, and the Capacity to Be Vulnerable

Reintegration required families to renegotiate roles influenced by military hierarchy, adaptation to absence, and evolving expectations upon the veteran's return, a process well documented in family systems and military transition literature (Gil‐Rivas et al. 2017; Knobloch and Theiss 2012; Riggs and Riggs 2011). Role flexibility, defined as the capacity for shared responsibility, openness to changing roles, and responsiveness to relational feedback, has been shown to foster trust and emotional safety in both military and civilian families (Hicks et al. 2024; Riggs and Riggs 2011). Notably, studies by McGaw et al. (2020) and Brennan et al. (2021) suggest that role strain can emerge when family members are pressured to maintain caregiving responsibilities or navigate ambiguous expectations, often resulting in altered dynamics and increased relational stress.

These findings align with systemic models that view reintegration as a family stress process (Gil‐Rivas et al. 2017) and with research indicating that military stress and PTSD symptoms impact partner and family functioning beyond the individual veteran (Brennan et al. 2021; McGaw et al. 2020). Going beyond these frameworks, this study emphasizes that role negotiation is fundamentally a process of relational trust, not merely a logistical or practical adjustment. This perspective echoes Knobloch and Theiss (2012), who highlight the importance of re‐negotiating authority and care as central to postdeployment adaptation. Veterans in this study reported experiencing mistrust when family systems failed to accommodate vulnerability or to distribute caregiving in ways that increased burden on others. Conversely, role flexibility fostered connection and restored confidence in the family's capacity to share responsibilities, reinforcing findings by Hicks et al. (2024) on the value of adaptive role negotiation for emotional safety.

This perspective enhances relational frameworks that address postdeployment role ambiguity and adaptation (Gil‐Rivas et al. 2017; Knobloch and Theiss 2012) and provides a context for integrating moral‐injury‐informed interpretations of trust rupture (Buechner 2020). Even in families that were materially supportive, trust remained fragile when internal meaning‐making, vulnerability, and relational safety were disrupted, consistent with findings from Sayers (2011) and McGaw et al. (2020) that highlight the ongoing impact of exclusion from prior household roles. This underscores that trust relies not only on external support but also on the family system's capacity to reorganize authority, roles, and care responsively. Research suggests that combat veterans often feel excluded from their previous household roles or like guests in their own homes (Brennan et al. 2021; Sayers 2011). Thus, a careful reassessment of pre‐deployment history and spousal dynamics is necessary to differentiate reintegration challenges from preexisting structural issues, as emphasized in recent work on military family adaptation (Gil‐Rivas et al. 2017).

### 4.5 Clinical Implications for Marriage and Family Therapy

Recognizing reintegration as a relational process instead of merely an individual treatment issue holds significant ramifications for marriage and family therapists assisting Black veterans and their families. This perspective is supported by a growing body of research advocating for systemic, contextually attuned approaches to military family care (Gewirtz et al. 2018; Hodgson and Lamson 2020; Sayers 2011). Consistent with these frameworks, trust is cultivated not through pressure to disclose emotions but through the co‐creation of relational safety, including sustained presence, emotional attunement, and role flexibility (Blow et al. 2015; Drew et al. 2022). Recent studies highlight the value of reframing silence and withdrawal as potentially protective regulatory strategies, rather than resistance (Collins and Tam 2024; Knobloch et al. 2023; Paley and Hajal 2022), and emphasize the importance of systemic signals such as tone, pacing, and persistence in conveying availability and support. Family therapy interventions that focus on renegotiating roles, shared responsibility, and openness to feedback align with evidence that adaptive role flexibility and collaborative problem‐solving foster trust and emotional safety during reintegration, especially when strict hierarchies or caregiving expectations make vulnerability difficult (Hicks et al. 2024; Lee et al. 2023; Sayers 2011).

Cultural attunement and systemic advocacy are also central to effective clinical work with Black veterans and their families. Research consistently demonstrates that racial identity shapes reintegration as both a source of resilience and a source of strain (Carlson et al. 2018; Dorsey Holliman et al. 2018; Nillni et al. 2023), highlighting the necessity of situating clinical concerns within broader racialized and sociopolitical contexts (Sharifian et al. 2024; Whealin et al. 2024). Discriminatory stress is associated with poorer mental health consequences among veterans of color (McClendon et al. 2021; Winters et al. 2023), while cultural resources such as spirituality, intergenerational narratives, and community ties can buffer distress and facilitate recovery (Carlson et al. 2018; Dorsey Holliman et al. 2018; Goodman et al. 2018). Marriage and family therapists are uniquely positioned to integrate these dynamics into treatment and to support families in navigating institutional barriers through advocacy, collaboration with VA systems, and connection to culturally responsive resources and community supports (Golojuch et al. 2025; Hodgson and Lamson 2020; Lee et al. 2023).

### 4.6 Limitations and Future Directions

This study has several limitations. The sample, comprised of 23 Black veterans primarily recruited through Veterans Affairs programs in New York City, may constrain the transferability of findings to other regions or veterans outside formal care systems. Reliance on community referrals and established trust may also have introduced selection bias. Persistent mistrust, confidentiality concerns, and institutional barriers among African American veterans (Barrett et al. 2017) likely shaped participation, further narrowing the diversity of reintegration experiences. To address these issues, future research should develop and assess recruitment strategies that directly confront mistrust, such as partnerships with veteran‐led organizations and culturally responsive outreach (Castro et al. 2015).

This study did not prioritize or systematically account for time since each veteran's reintegration, nor did it collect data on the number of deployment cycles completed prior to reintegration. These factors likely shape how veterans recall and interpret their experiences, as those with a longer period since return or multiple deployments may view trust, connection, and adjustment differently than those who returned more recently or experienced a single deployment (Adler et al. 2011). Future research should more deliberately consider both the timing of reintegration and deployment history to deepen the understanding of how these variables influence veterans' postdeployment experiences.

Methodological considerations related to race are also significant. Recruitment, participation, and disclosure among African American participants are influenced by historical mistrust, researcher identity, and institutional affiliation (Gibson and Abrams 2003). These factors, along with trust and perceived power dynamics during interviews, may have shaped participant narratives. Ongoing use of culturally responsive, trust‐building strategies to address past institutional harm remains essential to enhance transparency and participant comfort (Henderson et al. 2024). Additionally, the analytic process used a single coder, which may introduce interpretive bias and limit the inclusion of multiple analytic perspectives; credibility was supported through member checking peer debriefing, and an audit trail (Birt et al. 2016).

Limited participation by family members constrained examination of reintegration as an interactional family process. Although dyadic interviews were allowed to protect veterans' psychological safety and autonomy, logistical and relational barriers further restricted their involvement, reducing opportunities for triangulation and real‐time observation of family dynamics. Dyadic interviews offer valuable insights into how partners co‐construct meaning (Szulc and King 2022). Additionally, participants represented a variety of living arrangements, including independent and family‐based settings; however, the study did not explicitly examine how reintegration experiences may differ across these settings. As a result, variation in relational processes shaped by distinct living contexts was not fully captured. Future studies should consider flexible designs that integrate both parallel and dyadic approaches, supporting systemic analysis while upholding trauma‐informed safeguards.

All participating veterans reported PTSD diagnoses, resulting in a diagnostically homogenous sample. Reintegration processes may differ for veterans without PTSD or across varying levels of symptom severity and comorbidity. Future studies should include veterans with more diverse mental health profiles to clarify how relational trust, communication, and role negotiation vary across diagnostic contexts.

Finally, data collection occurred in 2020 during the COVID‐19 pandemic and heightened national awareness of anti‐Black violence following the murder of George Floyd. This sociopolitical context likely intensified historical trauma and institutional mistrust in Black communities (Crooks et al. 2021). Ongoing structural racism and racialized stress within African American family systems (George et al. 2014) may have shaped participant narratives, amplifying vigilance, guarded communication, and relational strain. Future research should include longitudinal studies across diverse sociopolitical contexts to distinguish enduring features of reintegration from those shaped by historical events. Moving beyond descriptive analysis, future research should evaluate systemic interventions that support Black veterans' reintegration and identify optimal points for therapeutic engagement. Intervention‐focused, longitudinal, and community‐partnered designs will strengthen both clinical relevance and ethical rigor.

## Funding

The author has nothing to report.

## Supporting information

Supporting File:

## Data Availability

The data that support the findings of this study are available on request from the corresponding author. The data are not publicly available due to privacy or ethical restrictions.
