# Supplementary material for: Building Trust and Connection: A Family Systems Perspective on Black Veteran Reintegration
Source: J Marital Fam Ther. 2026 May 12;52:e70144. doi: 10.1111/jmft.70144 (PMC13162192; doi:10.1111/jmft.70144)
Supplement: Supplementary file 1 — Supporting File: [file JMFT-52-0-s001.docx]

**Appendix A: Building Trust and Connection Interview Questions**

The questions from the interview are as follows. Participants were encouraged to elaborate on their responses to each question. When more information was needed, probe questions were employed.

**Introduction:** I am really interested in the reintegration process you guys have been through. As a result, I will ask about your experiences with reintegration and racial identity as a Black military family.

Tell me about your reintegration experiences (*directed to the veterans and family members*).

- **Prompts:** What ways do you think your experiences during deployment affect your reintegration (*directed to the veterans and family members*)?

o How have you noticed the other person shift or respond to reintegration? (*Probe: How have things changed or remained the same in the dynamics since your return? Give examples. What has felt different in your relationship since reintegration?*)

o How have you experienced support from each other during this reintegration process? Provide examples. *(Probe: What helps you feel supported? What makes it easier or harder to lean on someone? What does feeling connected mean to you?)*

- **Prompts:** When did you first become aware of your race, and how does it shape [who you are and] your roles in your family (e.g., partner, parent)?”

o How do you understand your racial and cultural identities as helping to support the experiences you have described? (*i.e., examples of racial/cultural values*)

o How do you feel your race impacted your military experiences? (*Probe: How has your race been addressed/ignored/treated in your military branch? How do you see the influence of your race on your military experiences?)*

- **Prompts:** What are some of the identities that you identify with since you’ve returned from the military?

o As a Black combat veteran with PTSD, are any of those identities most important or salient to your experiences of yourself and your reintegration processes? *(probe: other identities that the veteran has mentioned throughout the interview)*

**Conclusion:** Is there anything else you’d like to share about reintegration, racial and cultural identities, and your family processes?
